# Blockade of retinal or cortical activity does not prevent the development of callosal patches normally associated with ocular dominance columns in primary visual cortex

**DOI:** 10.1017/S0952523821000110

**Published:** 2021-08-23

**Authors:** Hsueh Chung Lu, Robyn J. Laing, Jaime F. Olavarria

**Affiliations:** Department of Psychology, and Behavior and Neuroscience Program, University of Washington, Seattle, Washington USA

**Keywords:** Long Evans rats, interhemispheric connections, tetrodotoxin, columnar organization, segregation

## Abstract

Callosal patches in primary visual cortex of Long Evans rats, normally associated with ocular dominance columns, emerge by postnatal day 10 (P10), but they do not form in rats monocularly enucleated a few days before P10. We investigated whether we could replicate the results of monocular enucleation by using tetrodotoxin (TTX) to block neural activity in one eye, or in primary visual cortex. Animals received daily intravitreal (P6–P9) or intracortical (P7–P9) injections of TTX, and our physiological evaluation of the efficacy of these injections indicated that the blockade induced by a single injection lasted at least 24 h. Four weeks later, the patterns of callosal connections in one hemisphere were revealed after multiple injections of horseradish peroxidase in the other hemisphere. We found that in rats receiving either intravitreal or cortical injections of TTX, the patterns of callosal patches analyzed in tangential sections from the flattened cortex were not significantly different from the pattern in normal rats. Our findings, therefore, suggest that the effects of monocular enucleation on the distribution of callosal connections are not due to the resulting imbalance of afferent ganglion cell activity, and that factors other than neural activity are likely involved.

## Introduction

Visual callosal connections in several species have proven to be a useful model for investigating the role of sensory input in the development of cortical connections (Cusick & Lund, 1982; Olavarria et al., 1987; reviewed in Olavarria, 2002; Restani & Caleo, 2016). However, most of these early studies do not specifically examine the role of neural activity in this process. Evidence that neural activity plays an important role in the development of visual callosal connections comes from studies examining the effect of reducing excitability of callosal neurons *via* overexpression of Kir2.1, a hyperpolarizing inward-rectifying potassium channel (Mizuno et al., 2007, 2010). In general, these studies analyze the role of neural activity on the radial growth and arborization of callosal axons, but do not inform about the role of neural activity in the establishment of the tangential distribution of callosal connections across cortex.

It was recently reported that callosal connections in primary visual cortex (V1) of Long Evans rats segregate into patches that overlap with ipsilateral ocular dominance columns (ODCs) (Laing et al., 2015). A previous study suggested that a critical period for the specification and development of callosal patches starts by P6 (Olavarria et al., 1987). Consistent with this, in the present study, we observed that monocular enucleation at P8 prevents the formation of callosal patches in both hemispheres. Callosal patches are normally visible at P10, age at which they become resistant to changes induced by monocular and binocular enucleation (Olavarria et al., 2021). These observations suggest that segregation of callosal patches requires some form of retinal input during a relatively short period prior to P10. To investigate whether retinal activity is necessary during this period for the specification and development of callosal patches, we carried out daily (P6–P9) monocular intravitreal injections of tetrodotoxin (TTX), and subsequently examined the pattern of callosal connections in V1 following multiple intracortical injections of the tracer horseradish peroxidase (HRP) in the opposite hemisphere. The patterns of callosal connections ipsilateral and contralateral to the intravitreal TTX injections were examined in separate groups. To test whether cortical neuronal activity plays a role in callosal patch development, in an additional group of rats we examined the HRP-labeled pattern of callosal connections in one hemisphere after daily (P7–P9) intracortical TTX injections into the same hemisphere. We found that in rats receiving either intravitreal or cortical injections of TTX, the patterns of callosal patches were not significantly different from the pattern in control rats. These results provide evidence that neither retinal nor cortical activity play a significant role in the specification and development of V1 callosal patches before P10.

## Materials and methods

Long Evans pigmented rats (*Rattus norvegicus)* were used and procedures were performed according to protocols approved by the Institutional Animal Care and Use Committee at the University of Washington, and are in accordance with the animal care guidelines of the National Institutes of Health, USA.

Daily (P6–P9) intravitreal or intracortical (P7–P9) injections of TTX were performed under isoflurane anesthesia (2.5% in air). To block activity in the retina of one eye, the eyelid was opened and TTX in citrate buffer (1 mg/ml; 0.0625 *μ*l, Affix Scientific, Fremont, CA) was pressure injected through a glass micropipette (50–100 *μ*m tip diameter) into the posterior chamber of the eye, 1.5–2.0 mm posterior to the corneal limbus, at a depth of 1.5–2.0 mm. To block cortical activity in V1, the injection of TTX (0.0625 *μ*l) was placed near the lateral border of V1 (4.0 mm lateral to the midline suture, 1.5 mm anterior to the lambda suture). In a separate group of pups, daily (P7–P9) intracortical injections of citrate buffer (0.0625 *μ*l) were administered. At P28, callosal connections were labeled following about 20 multiple intracortical injections of HRP (Sigma Co., 30% in saline; total volume = 4.0 *μ*l) delivered into the opposite occipital cortex through glass micropipettes (50–100 *μ*m tip diameter) over an area extending from ~2.0 to 7.0 mm lateral to the midline, and 0.0–6.0 mm anterior to lambda suture. To assess the condition of the retino-geniculate projection after the series of intravitreal TTX injections, 1.0 *μ*l of 30% HRP in saline was injected into the same eye.

**Fig. 1.**
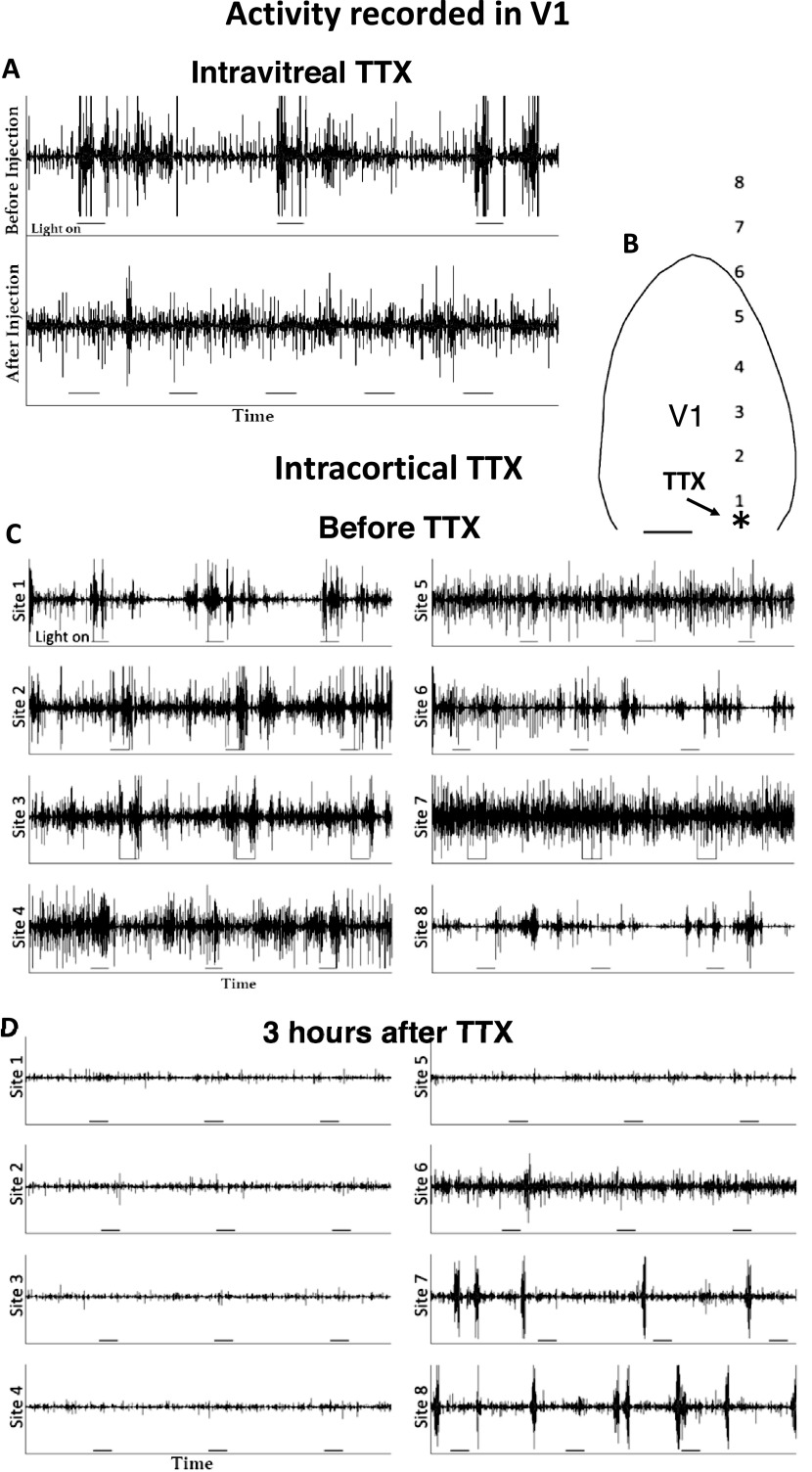
Assessment of the effectiveness of intravitreal and intracortical injections of TTX. (**A**) Intravitreal injections of TTX. Upper panel illustrates activity recorded before the TTX injection. It shows bursts of activity correlated with periods when the light stimulus was turned on (horizontal line segment underneath activity trace). Lower panel shows that 15 min after the TTX injection, only spontaneous activity uncorrelated to the light stimuli was recorded. (**B**) Intracortical TTX injection. The arrow points to the location of the TTX injection (asterisk), located 0.5 mm anterior to the lambda suture, and 3.5 mm lateral to the brain midline. The black contour corresponds to the border of V1. Lateral to the right, posterior is down. The numbers indicate eight recording sites separated by about 1.0 mm from each other. Scale bar = 1.0 mm. Activity was recorded by stimulating the contralateral eye. (**C**) Activity recorded before the intracortical TTX injection. The tracings show activity recorded at the sites indicated. (**D**) Activity recorded 3 h after the injection. The tracings show recordings at the sites indicated. Line segments under the recordings indicate light on.

Electrophysiology was performed as previously described (Olavarria et al., 2021). Briefly, using standard electrophysiological techniques, visually evoked responses were recorded in V1 of an additional group of rats (3 weeks of age) to assess the efficacy of the intravitreal and intracortical TTX injections (Fig. 1). Recordings were performed under urethane (1200 mg/kg i.p.) and ketamine (0.1 mg/kg) anesthesia. After exposing the occipital cortex, multiunit activity was recorded with glass-insulated tungsten electrodes (1–2 MΩ; FHC, Bowdoin, ME) at depths of 500–600 *μ*m. Signals were filtered, amplified, and stored on a computer for later analysis. Full field visual stimuli were presented through custom-made eye covers housing a white LED for each eye. A custom-made remote control simultaneously sent a signal to one LED as well as the electrophysiology interface board, allowing visually evoked responses to be time-locked to stimulation. Each eye was stimulated separately while the nonstimulated eye was covered.

Two days after the intravitreal and intracortical injections of HRP, animals were deeply anesthetized with pentobarbital sodium (100 mg/kg i.p.) and perfused with 0.9% saline followed by 4% paraformaldehyde in 0.1 m phosphate buffer (pH 7.4). The hemisphere opposite to the one injected with HRP was flattened, frozen, and sectioned into tangential sections (60 *μ*m thickness), which, together with coronal sections trough the lateral geniculate nucleus (LGN) and midbrain, were processed for HRP histochemistry (Mesulam, 1978). Data on the callosal patches in normally reared rats from our previous study (Olavarria et al., 2021) were available for comparison.

**Fig. 2.**
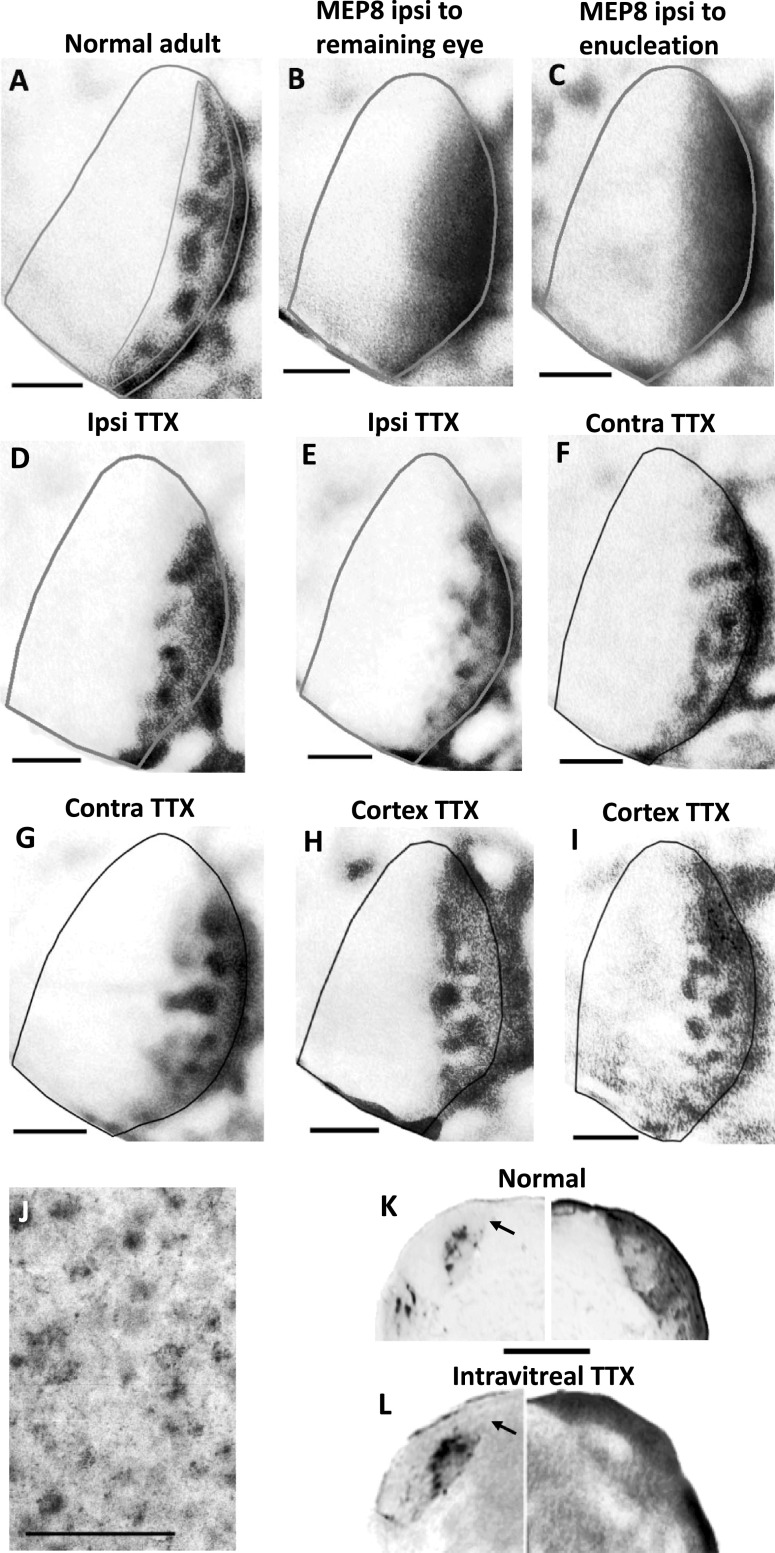
(**A**) Pattern of HRP-labeled callosal connections in normal adult rat (modified from Olavarria et al., 2021). The thin line delineates the central segment of V1 and illustrates the region used for analysis in all cases. This region does not include the band of callosal labeling at the 17/18a border. (**B,C**) Patterns of callosal connections ipsilateral to the remaining eye (**B**) and ipsilateral to the enucleation (**C**) in rats monocularly enucleated at P8. (**D,E**) Patterns of callosal connections in the hemisphere ipsilateral to the intravitreal injection of TTX. (**F,G**) Patterns of callosal connections in the hemisphere contralateral to the intravitreal injection of TTX. (**H,I)** Patterns of callosal connections in hemispheres injected with TTX. In **A–I**, note that callosal connections segregate into distinct patches in the central segment in all cases. Lateral is to the right, posterior is down. (**J**) Magnified view of cells in V1 labeled retrogradely with HRP in the case shown in (**B**). (**K**) HRP labeling pattern in the LGN of a normal adult rat showing ipsilateral (left) and contralateral (right) eye projections (modified from Olavarria et al., 2021). (**L**) HRP labeling in the LGN ipsilateral (left) and contralateral (right) to the intravitreal injections of TTX followed by injections of HRP. Arrows in **K** and **L** indicate dorsomedial region of the LGN ipsilateral to the HRP injections that remains unlabeled in both normal and TTX injected rats, suggesting that the intravitreal injection of TTX did not produce an expansion of the ipsilateral retino-geniculate projection into this region, as it occurs for the ipsilateral projection from the remaining eye in rats monocularly enucleated at P7 (Olavarria et al., 2021). Scale bar in **J** = 100 *μ*m, other scale bars = 1.0 mm.

Prior to histochemical processing, tangential cortical sections were digitally scanned to identify the V1 border, as revealed by the myelination pattern of V1 (Laing et al., 2015; Olavarria et al., 2021). The digitized images of myelin density and HRP labeling patterns were aligned with each other in Adobe Photoshop CS5 (Adobe Systems, Inc., San Jose, CA), using the border of V1, the edges of sections, and radial blood vessels as fiducial markers. In all images, noise and enhancement filters were applied to the entire images. The degree of patchiness (expressed as patch index) in the distribution of HRP labeling in the central segment of V1 (CS, outlined in Fig. 2A) was calculated in ImageJ (ver 1.50e) by measuring the s.d. of the gray value distributions: images with large variations in labeling density (i.e., high patch index) contain a wide range of gray values and have large s.d., whereas images with uniform distributions (i.e., low patch index) have a more restricted range of gray values and smaller s.d. ImageJ was also used to measure the areas of V1 and of a thresholded version of the callosal labeling in the CS of V1. These values were used to calculate the % of V1 occupied by the callosal region in the CS of V1. Measurements of the HRP-labeled area did not include the band of callosal connections at the border between areas 17 and 18a. High-magnification images were obtained using a DMR Leica microscope coupled to a Leica DC 300F digital camera. The Student *t*-test was used for comparing two groups (significance set at *P* < 0.05). ANOVA with Bonferroni posttest (significance set at *P* < 0.05) was used for multiple comparisons between groups.

## Results

The efficacy of the TTX injections was evaluated by recording visually evoked responses in V1 both before and after the intravitreal or intracortical injections of TTX. Three-week-old rats were used because visual evoked cortical responses are less reliable in newborn pups than in older rats (Fagiolini et al., 1994; Hanganu et al., 2006). We tested both light evoked responses and pupillary responses in older rats using the same dosage of TTX used in pups under the assumption that if the TTX injections had an effect in older and larger rats, they would also have an effect in a smaller animal. After recording reliable responses in V1 driven by the contralateral eye, TTX was injected either in the stimulated eye, or into the exposed V1, and recording resumed 15 min later to allow TTX to take effect. The pupillary response to light was not observed 15 min after an intravitreal injection of TTX.

Fig. 1A illustrates the evoked responses recorded from the same V1 site both before and 15 min after a TTX injection in the eye contralateral to the recorded hemisphere. The top panel shows that before the TTX injection, light stimuli elicited vigorous responses from a site in the contralateral binocular region of V1. Similar responses were observed after stimulating the ipsilateral eye. The bottom panel shows that 15 min after TTX injection, only spontaneous activity uncorrelated to the light stimuli was observed. Twenty hours after the TTX injection, a pupillary response to light was not observed in this animal, but the response returned to normal 28 h after the injection, suggesting that the blockade of retinal activity persists for approximately 1 day.

The intracortical TTX injection was placed 3.5 mm lateral to the midline and 0.5 mm in front of the lambda suture to allow the recording of evoked visual activity at eight sites separated by about 1.0 mm from each other and progressively more anterior relative to the injection site (see numbered sites in Fig. 1B). Fig. 1C shows that before the TTX injection, light stimuli elicited vigorous responses from sites 1–4 in the contralateral V1. The correlation between response and stimulus was less evident or absent for site 5, close to the anterior border of V1, and for sites 6–8 located beyond V1. Fig. 1D shows the responses 3 h after the TTX injection. Virtually all activity was blocked in sites 1–5, whereas activity uncorrelated to the visual stimulation reappeared beyond V1, in sites 6–8. Similar patterns of evoked responses were observed 15 min, as well as 21 h, after the TTX injection (data not shown). Taken together, these results indicate that our regime of daily injection of TTX blocked neural activity continuously.

In rats receiving intravitreal injections of TTX, we tested the possibility that absence of evoked cortical responses was due to retinal damage secondary to the TTX injections. We injected HRP into the eye that had been injected with TTX (see section “Materials and methods”) and examined the pattern of labeling in the LGN. We found that the ipsilateral (left in Fig. 2L) and contralateral (right in Fig. 2L) retino-geniculate projections from TTX injected eyes were similar to the respective projections in control animals (left and right in Fig. 2K [modified from Olavarria et al., 2021], respectively). In particular, arrows in Fig. 2K and 2L indicate the dorsomedial region of the LGN ipsilateral to the HRP injections that remains unlabeled in both normal and TTX injected rats, suggesting that the intravitreal injection of TTX did not produce an expansion of the ipsilateral retino-geniculate projection into this region, as it occurs for the ipsilateral projection from the remaining eye in rats monocularly enucleated at P7 (Olavarria et al., 2021). These observations indicate, on the one hand, that retinal damage was largely avoided, and suggest, on the other hand, that intraocular TTX injections do not affect the retino-geniculate projections in obvious ways.

**Fig. 3.**
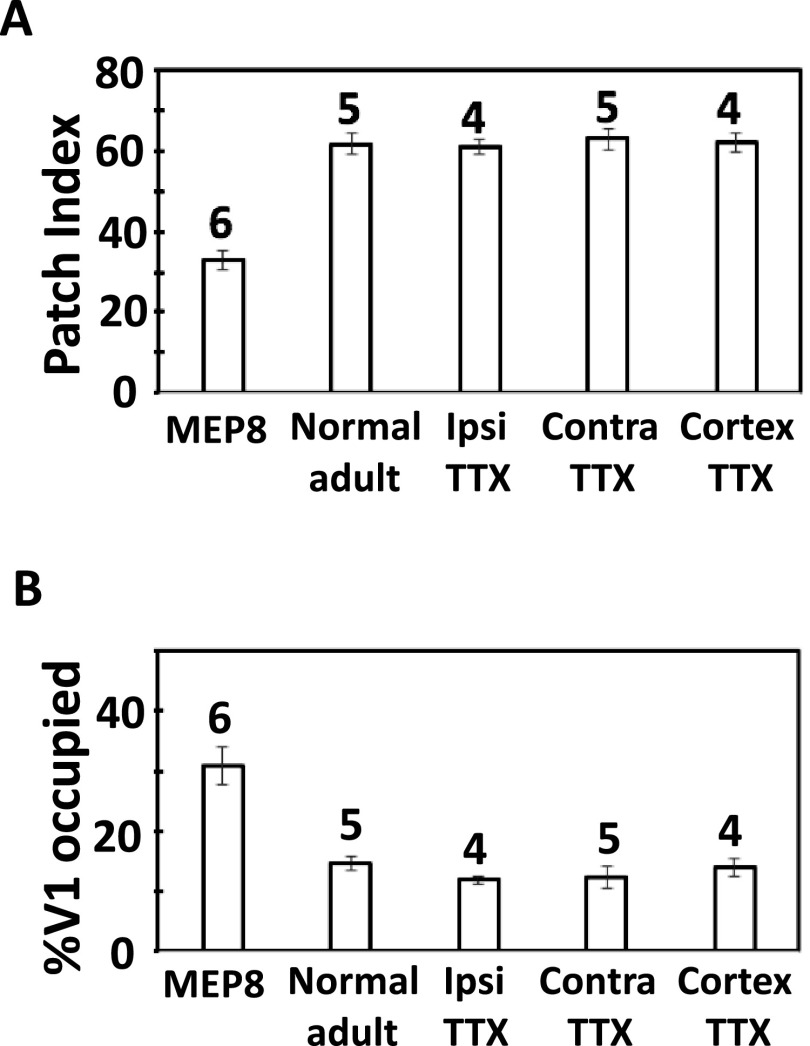
(**A**) Comparison of patch indices in the central segment in MEP8, normal adult, in the hemisphere ipsilateral (Ipsi TTX) and contralateral (Contra TTX) to the intravitreal injection of TTX, and in the hemisphere injected with TTX (Cortex TTX). (**B**) Comparison of the %V1 occupied by callosal patches in the central segment in MEP8, normal adult, in the hemisphere ipsilateral (Ipsi TTX) and contralateral (Contra TTX) to the intravitreal injection of TTX, and in the hemisphere injected with TTX (Cortex TTX). Numbers above the bars indicate number of animals in each group. Error bars indicate s.e.m. MEP8, monocular enucleation at P8.

The tangential pattern of callosal connections in V1 of normal adult rats, illustrated in Fig. 2A (modified from Olavarria et al., 2021), has been described and analyzed previously (Olavarria et al., 2021). It consists of a band of callosal connections, 0.2–0.3 mm wide adjacent to the lateral border of V1 and, further medially, a number of densely labeled patches occupying the CS (outlined in Fig. 2A). The patches typically vary in size and number. On average, the patch index for callosal connection in the CS of normal adult rats is 61.77 (s.e.m. = 2.84, *n* = 5), and the % of V1 occupied by callosal patches in V1 in normal adult is 14.67 (s.e.m. = 1.15, *n* = 5) (Fig. 3A and 3B, respectively). In contrast, in rats monocularly enucleated at P8, we found that callosal connections do not aggregate into patches in either hemisphere. Instead, they are homogeneously distributed over the lateral half of V1 (Fig. 2B and 2C). Fig. 2J shows cells retrogradely labeled with HRP from the cases shown in Fig. 2B. Combined analysis of both hemispheres in rats monocularly enucleated at P8 rats showed that, on average, the patch index was 32.96 (s.e.m. = 2.40, *n* = 6; Fig. 3A), and callosal connection in the CS occupied 30.72% (s.e.m. = 3.13, *n* = 6) of the V1 area (Fig. 3B). These values are significantly different (*P* > 0.05) from the respective values in normal adult rats.

In rats that received intravitreal injections of TTX in one eye, we found that callosal connections in the CS formed patches in both the hemisphere ipsilateral (Fig. 2D and 2E) and contralateral (Fig. 2F and 2G) to the injected eye. The averages patch indices for the ipsilateral (61.12, s.e.m. = 1.84, *n* = 4) and contralateral (62.894, s.e.m. = 2.62, *n* = 5) hemispheres where not significantly different (*P* > 0.05) from each other, nor from the patch index in normal rats (61.77, s.e.m. = 2.84, *n* = 5; Fig. 3A). Similarly, the % of V1 occupied by callosal patches in the CS ipsilateral (11.91, s.e.m. = 0.65, *n* = 4) and contralateral (12.33, s.e.m. = 1.84, *n* = 5) hemispheres where not significantly different (*P* > 0.05) from each other, nor from the % of V1 occupied by callosal patches in normal rats (14.67, s.e.m. = 1.15, *n* = 5) (Fig. 3B).

We also observed callosal patches in the CS of hemispheres injected with TTX (Fig. 2H and 2I), and, again, the values for patch index (62.15, s.e.m. = 2.39, *n* = 4) and % of V1 occupied by callosal connections in the CS (13.89, s.e.m. = 1.42, *n* = 4) were not significantly different (*P* > 0.05) from each other, nor from the respective values for patch index and % of V1 occupied by callosal connections in normal rats (Fig. 3A and 3B). Finally, as in normal rats, in all groups of animals studied, callosal connections accumulated forming a band at the lateral border of V1 (Fig. 2). In summary, our results do not reveal a significant effect of either intravitreal or intracortical injections of TTX on the development of callosal patches in V1. Likewise, the values for patch index (61.71, s.e.m. = 0.76, *n* = 3) and %V1 occupied (15.30, s.e.m. = 0.60, *n* = 3) following intracortical injections of citrate buffer placed approximately at the location chosen for the intracortical TTX injection (3.5 mm lateral to the midline and 0.5 mm in front of the lambda suture) were not significantly different (*P* > 0.05) from the respective values for patch index and % of V1 occupied by callosal connections in normal rats.

## Discussion

We found that chronic blockade of retinal or cortical activity induced by either monocular or intracortical injections of TTX in the days before P10 does not have the same effect on callosal development as monocular enucleation during the same period. Instead of a homogeneous distribution of callosal connections in the lateral half of V1, we observed that callosal connections form patches similar to those observed in normal animals. Our failure to replicate the anomalies in the callosal pattern produced by monocular enucleation could be due to incomplete blockade of retinal or cortical activity. This is unlikely because our electrophysiological recordings from V1 unequivocally showed that single injections of the TTX dose used in rat pups effectively blocked evoked retinal activity and all cortical activity for about 24 h in 3-week-old rats. Moreover, the results obtained were similar in all groups studied. HRP injections into the eye injected with TTX revealed patterns of LGN labeling similar to those observed in normal animals, indicating that transport by nerve fibers was adequate and that retinal lesions were avoided. We are therefore confident that our results are not due to incomplete or inconsistent blockade of retinal or cortical activity. Finally, our results are consistent with a previous study reporting that daily monocular injections of TTX failed to induce the formation of an anomalous callosal band observed running rostrocaudally along the middle of V1 in rats monocularly enucleated during the first days of life (Chang et al., 1995).

It is also possible that activity relayed by projections from the eye not injected with TTX is able to specify the development of callosal patches in at least one hemisphere. However, our intracortical injections of TTX provide evidence that this is not the case because activity relayed by both eyes to the TTX injected cortex was completely blocked, but callosal patches nevertheless developed as in normal rats.

Our findings therefore suggest that the changes in the tangential distribution of callosal connections brought about by monocular enucleation are not due to the resulting imbalance of afferent ganglion cell activity. However, previous studies provide evidence that reduction of activity in the retino-geniculo-cortical pathway influences the radial growth and arborization of callosal axons in visual cortex (Mizuno et al., 2007, 2010). Thus, it is possible that mechanisms depending on neural activity guide the radial development of callosal connections, while mechanism less dependent on neural activity preferentially guide the specification and establishment of the tangential pattern of callosal connections across visual cortex, including callosal patches. Another aspect of the tangential organization of callosal connections is the manner in which callosal fibers in V1 link topographically corresponding loci in both hemispheres that are not symmetric with respect to the brain midline (Lewis & Olavarria, 1995). This callosal map develops by P6 (Olavarria & Hiroi, 2003), and previous studies showed that the topography of callosal linkages is not altered by blocking retinal waves during the first postnatal week (Olavarria et al., 2020), or when *N*-methyl-d-aspartate receptors (NMDARs) are blocked from P4 to P8 (Olavarria et al. 2008), suggesting that neural activity mediated by NMDARs is not necessary for the establishment of the callosal map.

In Long Evans rats, callosal patches co-localize with ipsilateral ODCs in the CS (Laing et al., 2015), and both ODCs and callosal patches are visible by P10 (Olavarria et al., 2021). It is possible that common mechanisms guide the development of ODCs and callosal patches, or that the development of one structure specifies the development of the other. In rats monocularly enucleated at P8, we observed that callosal connections in both hemispheres are homogeneously distributed in the lateral half of V1 without forming patches. Similarly, in rats monocularly enucleated at P7, both the ipsilateral and contralateral retino-geniculo-cortical projections are also homogeneously distributed over large areas of V1 without forming ODCs (Olavarria et al., 2021). Moreover, the ipsilateral retino-LGN projection expands and invades the dorsomedial region of the LGN (Manford et al., 1984; Hayakawa & Kawasaki, 2010; Olavarria et al., 2021), region that remains free of label after ipsilateral eye tracer injections in normal rats and in rats that had one eye injected with TTX (arrows in Fig. 2K and 2L). This apparent lack of effect of our intraocular TTX injections on the retino-geniculate projections is consistent with studies reporting modest or no effect of intravitreal injections of TTX on geniculate layer formation and segregation of eye specific retino-geniculate projections (Casagrande & Condo, 1988; Cook et al., 1999; Hayakawa & Kawasaki, 2010). Thus, it remains possible that blockade of retinal and cortical activity in rats does not interfere with the segregation of geniculo-cortical projections into ODCs in rats, and that these, in turn, specify the development of associated callosal patches in V1. Further studies are needed to investigate a possible link between the development of ODCs and associated callosal patches, and the role that factors other than neural activity may play in this process.
